# Single fraction radiosurgery using Rapid Arc for treatment of intracranial targets

**DOI:** 10.1186/1748-717X-5-77

**Published:** 2010-09-13

**Authors:** Hendrik A Wolff, Daniela M Wagner, Hans Christiansen, Clemens F Hess, Hilke Vorwerk

**Affiliations:** 1 Department of Radiotherapy and Radiooncology, Universitätsmedizin Göttingen, Germany

## Abstract

**Background:**

Stereotactic-Radio-Surgery (SRS) using Conformal-Arc-Therapy (CAT) is a well established irradiation technique for treatment of intracranial targets. Although small safety margins are required because of very high accuracy of patient positioning and exact online localisation, there are still disadvantages like long treatment time, high number of monitor units (MU) and covering of noncircular targets. This planning study analysed whether Rapid Arc (RA) with stereotactic localisation for single-fraction SRS can solve these problems.

**Methods:**

Ten consecutive patients were treated with Linac-based SRS. Eight patients had one or more brain metastases. The other patients presented a symptomatic vestibularis schwannoma and an atypic meningeoma. For all patients, two plans (CAT/RA) were calculated and analysed.

**Results:**

Conformity was higher for RA with additional larger low-dose areas. Furthermore, RA reduced the number of MU and the treatment time for all patients. Dose to organs at risk were equal or slightly higher using RA in comparison to CAT.

**Conclusions:**

RA provides a new alternative for single-fraction SRS irradiation combining advantages of short treatment time with lower number of MU and better conformity in addition to accuracy of stereotactic localisation in selected cases with uncomplicated clinical realization.

## Background

Stereotactic Radiosurgery (SRS) using Conformal Arc Therapy (CAT) is a well established and commonly used irradiation technique for applying high dose to the target while sparing dose to surrounding critical structures via steep dose gradient outside the lesion [1,2]. A very high accuracy of patient positioning and exact online localisation during treatment is required to diminish the safety margin between gross tumour volume (GTV) and planning target volume (PTV). However, there are still some disadvantages like long treatment time, a large number of monitor units (MU), and difficulties in covering of noncircular or ellipsoid targets.

In the past, conventional Intensity Modulated Radiotherapy (IMRT) was tested to resolve the difficulties in covering of noncircular or ellipsoid targets with mixed success but without solving all described problems as well in fractionated as in single fraction irradiation procedures [3-7].

In the next step, Rapid Arc (RA) - as an advanced development of IMRT - was explored effectually for hypo-fractionated irradiation of brain metastases or benign intracranial diseases [8-10]. The RA technology delivers an entire IMRT treatment in a single gantry rotation around the patient. Three dynamic parameters can be continuously varied to create IMRT dose distributions: The speed of rotation, beam shaping aperture, and delivery dose rate [11]. The variation of these three dynamic parameters is used to cover the planning target volume with clinical acceptable dose and to minimise the dose to organs at risk (OAR) and normal tissue. Because of the volumetric single arc, treatment time is very short compared to IMRT or CAT including excellent target covering, especially for complex and irregular lesions. For example, Clivio et al. [12] found that RA showed improvements in lowering the dose to the OAR and healthy tissue with uncompromised target coverage in irradiation of patients with anal cancer. In contrast, the volume of low dose areas of the normal tissue is higher in RA delivery, and should be considered for selection of application technique, especially for young patients.

However, RA has been evaluated for application of hypo-fractionated radiotherapy but not for single fraction radiosurgery, yet. A treatment composed of single fraction RA irradiation with stereotactic localisation could possibly unify advantages of both treatment techniques with accuracy of radiosurgery, shorter treatment time, and better coverage of targets in selected cases.

Thus, aim of the present study was to compare quality criteria of both techniques for ten patients with different intracranial targets with special reference to feasibility, critical structures, and target covering.

## Patients and Methods

Ten consecutive patients with macroscopic intracranial tumours were treated with Linac based SRS at our department from 11/2008 to 10/2009. Two patients were women, eight patients were men, and the median age was 61.4 years (range 44 to 76 years). Eight patients received irradiation because of one or more intracranial metastases of a primary peripheral tumour. Five of these presented 1 solitary, two 2 and one patient 4 brain metastases, which were included into one treatment target volume (GTV) for treatment planning and later analysis. One patient showed a symptomatic vestibularis schwannoma on the left side, and another patient was treated because of an atypic meningeoma in the left area of the clivus. Each patient was reviewed by a radiation oncologist and neuroradiologist before SRS to verify treatment eligibility. The presented consecutive 10 cases showed varieties in number of isocenters, shape, volume and distances to critical structures and were consciously selected to evaluate positive and negative factors for both treatment modality options (patient and lesion characteristics are summarized in table 1). All procedures were followed in accordance with the ethical standards of the responsible committee on human experimentation and with Helsinki Declaration of 1975, as revised in 2000.

**Table 1:** Patient characteristics

Pat.no	Gender	Age(years)	Diagnosis	Summated GTV (cm^3^)	Number of isocenters	Prescribed SRS dose (Gy)	Prescription isodose CAT/RA (%)	Distance to nearest OAR (cm)
1	M	58	1 metastasis	0.1	1	11.0	80/95	3.7
2	M	76	Vestibularis schwannoma	0.9	2	13.0	70/95	0.6
3	F	44	2 metastases	0.3	2	22.0	80/95	4.2
4	M	55	1 metastasis	8.4	1	18.0	80/95	1.2
5	M	61	1 metastasis	3.2	1	18.0	80/95	2.8
6	M	60	1 metastasis	0.1	1	24.0	80/95	3.4
7	F	64	1 metastasis	0.7	1	24.0	80/95	4.0
8	M	72	Atypic meningeoma	2.7	1	14.0	70/95	2.8
9	F	64	4 metastases	2.0	4	22.0	80/95	3.5
10	M	60	2 metastases	0.3	2	24.0	80/95	3.6

F: female, M: Male, GTV: Gross Tumour Volume, SRS: Stereotactic Radiosurgery, CAT: Conformal Arc Technique, RA: Rapid Arc, OAR: organ at risk

### Treatment planning

Lesions of each patient were evaluated on a 1.5 mm slice magnetic resonance imaging (MRI) scan with contrast medium (Gadolinium). For Conformal Arc planning, image data set was transferred to the planning workstation where the responsible radiation oncologist (same person H.A.W. for all ten cases with expertise in SRS) manually outlined the target volume and OAR on axial images using FastPlan (version 5.5.1, Varian Medical Systems, Palo Alto, CA, USA). The GTV for CAT was defined using the contrast-enhancing T1 weighted MRI. The GTV should, as commonly recommended, be covered with either the 80% isodose line for one isocenter or the 70% isodose line for two or more isocenters to minimize the maximum dose inside the GTV due to the overlapping of two or more round treatment fields outlined with the cones. To accomplish optimal target covering different cone-widths from 5 mm to 25 mm were tested during planning procedure for each isocenter to achieve best results. No additional expansion of the target volume was added. If one patient had two or more targets, all separate targets were combined to one GTV for posterior plan evaluation. Multiple arcs (different numbers and angles of beams) were designed to take the best advantage of decreasing the dose to OAR's and normal brain tissue.

In the next step, a high resolution computer tomography (CT) scan with 3 mm slices was performed with SOMATOM Balance (Siemens Medical Systems, Forchheim, Germany). For this examination, a customized bite block for later localisation during treatment procedure was prepared and patients were fixed on treatment couch with an individual thermoplastic mask. Afterwards, a simultaneous overlay in axial, coronal and sagittal reconstructions for MRI-CT fusion of both data sets was carried out to match the target volume on MRI scan with the localisation system using CT scan by the software FastPlan (see above).

Dose concept for each patient was assessed individually dependent on tumour entity, tumour volume and involved critical structures: Metastases were irradiated with a dose between 11 Gy and 24 Gy, whereas the patient with vestibularis schwannoma received a dose of 13 Gy. Dose concept for one patient with atypic meningeoma was calculated to 14 Gy. Photon energy was assessed to 6 MV for all plans.

For each patient another treatment plan using RA was calculated on the same CT/MRI scan. All RA plans were designed using a progressive resolution algorithm (PRO, version 8.2.23, Varian, Medical Systems, Helsinki, Finland). The dose distribution was calculated using the anisotropic analytical algorithm with a grid size of 0.2 cm × 0.2 cm × 0.2 cm (AAA, version 8.2.23, Varian Medical System, Helsinki, Finland). The AAA is a 3D pencil beam convolution/superposition algorithm that uses separate Monte Carlo derived modelling for primary photons, scattered extra-focal photons, and electrons scattered from the beam limiting devices [13,14]. The single arc treatment field was split in 177 control points. The modulation was achieved by delivering 177 control points. For each control point, the beam aperture as defined by Millennium 120 multi leaf collimator (MLC) (Varian Medical Systems, Palo Alto, CA, USA) changed with the gantry angle to deliver the intensity modulated dose to the patient. The dose rate was varied between 0 MU/min to a maximum of 800 MU/min and the gantry rotation between 0.0°/sec and a maximum of about 4.8°/sec. To minimise the contribution of tongue and groove effect during the treatment the collimator was rotated to 45°. All plans were generated using the Eclipse planning system (Version 8.5, Varian Medical Systems, Helsinki, Finland). The quality assurance of Rapid Arc treatment fields was conducted with the "I'mRT-MatriXX" (Scanditronix, Wellhöfer, Schwarzenbruck, Germany) and the Software: "OmniPro I'mRT" (version 1.5, Scanditronix, Wellhöfer, Schwarzenbruck, Germany). Only in one patient a full rotation was necessary to cover the GTV.

The GTV had to be covered by the 95% isodose line. According to the ICRU 50 report [15] the maximum dose should not exceed 107% of the prescribed dose. Organs at risk including the brainstem, chiasm, optical nerves, healthy brain and lenses were contoured manually on each single MRT slice for dose-volume-histogram (DVH) analysis. The dose to OAR was aimed to be as low as possible.

### Stereotactic Radiosurgery Treatment Procedure

All patients received single session Linac based SRS. Therefore, the patients were placed supine on the treatment couch as before during CT scan. In the next step, the previously constructed thermoplastic mask and the bite block with reflecting fiducials was attached to the patient. Patient position was registered by the reflecting fiducials and an in room camera system. The camera system was verified before patient setup. Due to the verification process the camera system saves the position of the linac based isocenter in the treatment room. The information about the treatment plan based isocenter was send to the camera system. The camera system displayed the shift between the isocenter defined by the bite block fiducials and the treatment plan based isocenter. After the alignment of both isocenters the patient is positioned exactly to the treatment plan based isocenter. The irradiation took place at a Varian 2300 C/D Clinac (Varian Medical Systems, Palo Alto, CA, USA) with fix cones for CAT. For RA treatment the patients can be localized within the isocenter via the same in room camera system before single arc irradiation.

### Dosimetric evaluation parameters and statistical analysis

Each treatment plan was evaluated with regard to target coverage, dose to OAR, treatment time, number of MU and irradiated normal tissue. PTV conformity index (CI) was reviewed according to the technique dependent standard constrains including commonly valid prescription doses for each technique as follows: For CAT plans, ratio of target volume covered by the 80% isodose line for one isocenter or the 70% isodose line for two or more isocenters divided by the total volume covered by that isodose line was calculated. For all RA plans the ratio of target volume covered by the 95% isodose line divided by the total volume covered by that isodose line was measured as usually recommended. The volume of the body irradiated with 2 Gy was calculated to assess low dose areas. The mean dose (D_mean_) to the healthy brain and the maximum dose (D_max,OAR_) to OAR and GTV were calculated and dosimetric results were compared for both irradiation techniques. The maximum dose (D_max,GTV_) was defined as the maximum dose value measured within the target volume. Analyses of inhomogeneity indices were not performed in detail because of intended incomparable results through the generated GTV for SRS using CAT with 70% or 80% isodose line for target covering with a D_max,GTV _up to 140% of the prescribed dose in comparison to maximum dose of 107% using RA according to ICRU report [15]. Because of these established, technique dependent constrains RA homogeneity indices were clearly better and would afford no reasonable information.

## Results

### GTV coverage and conformity

Mean volume of GTV was 0.8 cm^3^, median 1.78 cm^3^, minimum 0.1 cm^3 ^and maximum 8.4 cm^3^. Although conflicts existed in some plans resulting from the position of OAR's relative to the target volume (table 1), GTV coverage was 100% in both different treatment techniques for all patients. Thus, there was no need to crop dose to the GTVs, even for central target localisations like vestibularis schwannomas with small distance to the chiasm, brainstem or optical nerves.

**Figure 1:**
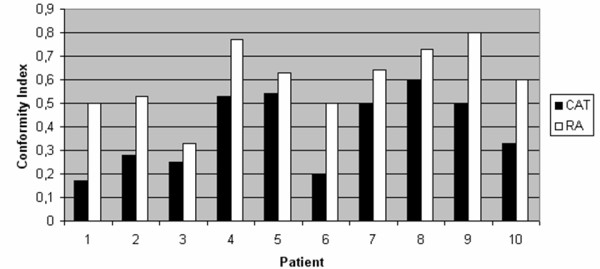
Diagram of Conformity Index for CAT (Conformal Arc Therapy) in black and for RA (Rapid Arc) in white

Conformity indices were clearly better for RA in all analysed GTV localisation and treatment volumes with a median of 0.56 compared to 0.37 for CAT (figure 1). Especially, irregularly formed tumours were framed more precise with the prescribed dose including less normal tissue or OAR in high dose area. Largest improvement was achieved in patient 1 with a factor of 2.94 (RA: 0.50; CAT: 0.17).

Although inhomogeneity index was not be analysed reasonable in detail (see above), the D_max _of GTV was reduced dramatically for all patients using RA (19.9 Gy vs. 24.4 Gy).

### Dose to organs at risk and normal tissue

**Figure 2:**
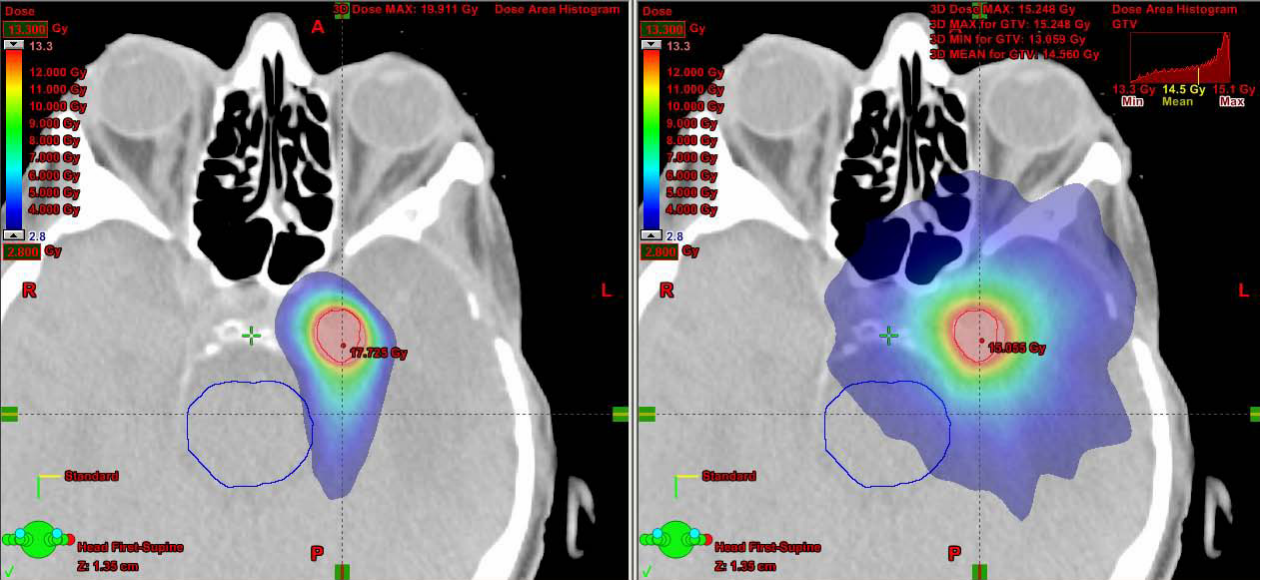
Comparison of representative dose distributions for conformal arc (left) and RapidArc (right) illustrating typical differences between both techniques in patient 8 treated because of an atypic meningeoma in the area of the clivus.

In general, the dose to OAR is very low. However, in 7 of 10 analysed patients RA achieved a better dose preservation of OAR in general comparison of all involved tissues. Only patient 3, 4 and 8 showed a predominantly better result for CAT (table 2). Two of these patients had peripheral brain metastases with a large distance to central OAR like brainstem, optical nerves or lenses (patients 3 and 4). The other patient, treated because of an atypic meningeoma in the area of the clivus, could be irradiated with constant lower doses at all OAR because of the steeper dose gradient using CAT. Patient associated dose distributions of both techniques were illustrated in (figure 2).

**Table 2:** Summary of Organs at risk

Patient No.	1		2		3		4		5		6		7		8		9		10	
**Technique**	**CAT**	**RA**	**CAT**	**RA**	**CAT**	**RA**	**CAT**	**RA**	**CAT**	**RA**	**CAT**	**RA**	**CAT**	**RA**	**CAT**	**RA**	**CAT**	**RA**	**CAT**	**RA**
**Healthy brain D_mean _[Gy]**	0.1	0.3	0.4	0.5	**0.6**	3	1.0	1.4	**2.1**	5.2	0.3	0.6	0.6	0.7	0.6	1	**1.3**	3.2	**0.5**	2.2
**V_2Gy _[cm^3^]**	**8.6**	53	**59.5**	104.8	**68.7**	860.3	**316.8**	394.4	**92.3**	145.7	**37.6**	99.9	100.8	105.7	**99.6**	367.6	**223.7**	1057	**71.4**	601
OAR D_max _[Gy]																				
**Lens left**	0.0	0.5	0.0	0.1	**0.1**	1.3	**0.0**	1.3	0.6	0.1	0.3	0.2	0.0	0.0	**0.0**	1.9	0.6	0.3	0.3	0.6
**Lens right**	0.0	0.4	0.0	0.1	**0.0**	1.4	**1.1**	2.5	0.6	0.0	0.0	0.1	0.0	0.0	0.0	**1.3**	0.0	0.3	0.3	0.6
Brainstem	0.4	1.5	**3.7**	6.9	**0.5**	7.1	**2.3**	3.4	0.8	0.7	0.5	0.8	0.9	0.1	4.9	4.9	2.0	1.2	**0.9**	2.1
**Chiasm**	0.0	0.9	**0.5**	3.8	**0.1**	3.8	**0.7**	2.5	0.6	0.0	0.0	0.4	0.0	0.1	**1.2**	4.2	1.4	1.7	**0.0**	1.7
**N. opticus right**	0.0	0.6	0.0	0.2	**0.2**	3.2	2.4	1.4	0.7	0.1	0.0	0.3	0.0	0.0	**0.3**	2.6	0.5	1.0	0.4	1.3
N. opticus left	0.0	0.9	0.5	0.3	**0.2**	3.7	0.0	0.9	0.8	0.1	0.5	0.3	0.0	0.0	**1.2**	4.6	1.4	1.0	0.5	1.4

Healthy brain (D_mean_), low-dose volume (V_2 Gy_) for all patients and both treatment techniques. The maximum dose to OAR is shown in Gy, the mean dose to healthy brain in Gy, and the low dose volume (volume which is irradiated with maximum of 2 Gy) in cm^3^.

OAR = organs at risk, CAT = conformal arc therapy, RA = Rapid Arc, D_mean _= mean dose, V_2 Gy _= volume irradiated with 2 Gy or higher, **Fat marked fields **indicate a benefit for this value

The D_mean _of the healthy brain tissue was lower using RA in all patients. In contrast, low-dose areas could be kept clearly smaller using CAT in all cases (table 2). In maximum, low-dose volume was up to 12.5 times smaller using CAT for treatment of 2 peripheral brain metastases in patient 3 compared to RA.

### Field Setup, Treatment Time and Monitor Units

**Figure 3:**
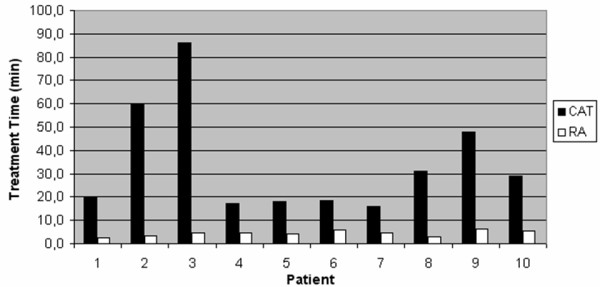
**Diagram of Treatment Time for CAT (Conformal Arc Therapy) in black and for RA (Rapid Arc) in white**. The treatment time does not include the setup of patient and setup between every single arc for CAT. The displayed treatment time is the time where the beam is on.

The number of arcs using CAT depended on the number of required isocenters, whereas for RA single isocenter planning was used. Patients with one isocenter were treated with 5 arcs in conformal therapy, whereas patients with two isocenters received 10 to 12 arcs. Additionally, patient 9 with four different isocenters was irradiated with 20 arcs. Treatment time for delivering prescribed dose was definitely longer in all CAT cases compared to single RA treatment (median time: 34.4 min vs. 4.5 min). Especially, for irradiation of patients with more than one isocenter, treatment time was ≥ 17 times longer using CAT (patients 2 and 3) (figure 3).

**Figure 4:**
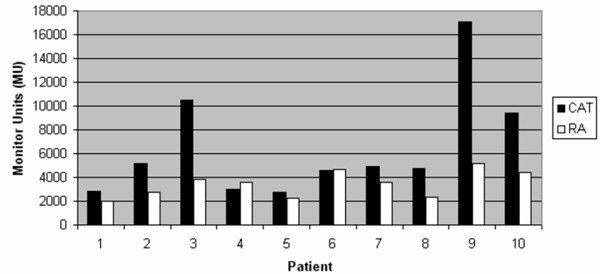
**Diagram of calculated Monitor Units (MU) for CAT (Conformal Arc Therapy) in black and RA (Rapid Arc) in white**. The MU for each single arc using CAT were summed up and displayed. For RA only one arc was used.

Furthermore, median number of MU was 6504 MU for CAT and 3455 MU for RA. In patient 6 with single peripheral metastasis the number of MU was nearly the same for both techniques (4618 MU (CAT) vs. 4663 MU (RA)), whereas for patient 4 CAT needed only 2964 MU compared to 3577 MU for RA for one single peripheral metastases. In all other cases, RA got along with obvious less number of MU (figure 4).

## Discussion

Our data show promising results analysing and implementing a new approach for delivering single fraction radiosurgery via RA with additional advantages in comparison to standard Conformal Arc application accuracy. Similar results for IMRT were shown before by Baumert et al. 2003 [16] by analyzing intensity modulated radiotherapy compared to conformal static arc therapy in treatment of meningioma of the skull base. In this work, IMRT was superior in PTV coverage with lower doses to the OAR admittedly in fractionated therapy regime, too. In another work from Wu et al. [6], results for treatment of intracranial lesions using IMRT were classified as superior to a 3D-conformal static technique and dynamic conformal arcs concerning dosimetric benefits for SRS. However, these studies showed positive results even without including the new benefits evolving through tested RA. In this context, Clark et al. [8] evaluated the relative plan quality of single-isocenter vs. multi-isocenter radiosurgical treatment of multiple central nervous system metastases for VMAT irradiation. In this planning study, plans were created using VMAT for treatment of simulated patients with three brain metastases. They concluded that radiosurgery for multiple targets using a single isocenter can be efficiently delivered, requiring less than one-half the beam time required for multiple isocenter set ups, too. In their opinion, VMAT radiosurgery will likely replace multi-isocenter techniques for linear accelerator-based treatment of multiple targets in the future. Furthermore, Lagerwaard et al. [10] used RA to plan and deliver whole-brain radiotherapy (WBRT) with a simultaneous integrated boost in patients with multiple brain metastases. In this study, RA plans showed excellent coverage of planning target volume for WBRT and PTV for the boost. These result led the authors to the conclusion that RA treatment planning and delivery of integrated plans of WBRT and boosts to multiple brain metastases is a rapid and accurate technique that has a higher conformity index than conventional summation of WBRT and radiosurgery boost.

**Figure 5:**
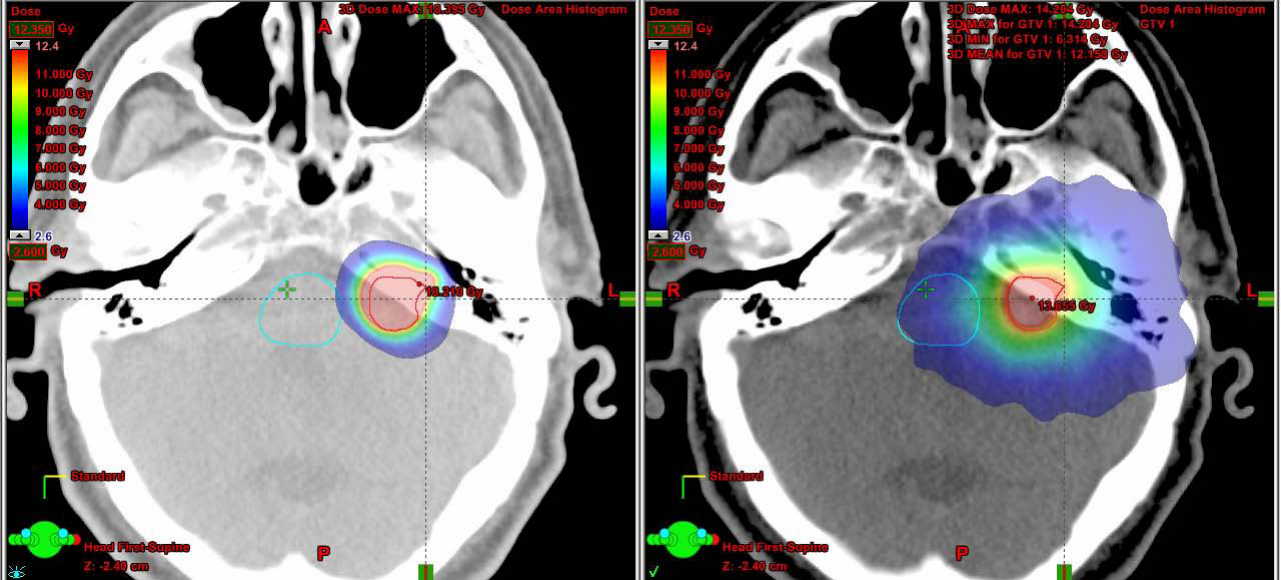
Comparison of representative dose distributions for conformal arc (left) and RapidArc (right) illustrating typical differences between both techniques in patient 2 treated because of a vestibularis schwannoma.

In comparison, our conformity results were also better for RA because of merely ellipsoid target shaping in CAT using cones with circular fields. Because of this fact, high-dose volumes could be kept significantly smaller with RA. In contrast, low-dose volume was clearly smaller using CAT in all patients. This fact could be expected because of rotation around the patient with continuous beam on time using RA with 177 control points compared to step and shoot irradiation using CAT with 5 to 20 arcs. Furthermore, the distance between beam shaping aperture and patient is higher for RA. For CAT, the cones minimise the distance between beam shaping aperture and patient and therefore generate steeper dose gradients. This result may play no decisive role when irradiation is indicated in palliative situation. However, whenever younger patients with longer estimated lifetime were analysed for irradiation, risk of development of a secondary tumour should be more weighted for final choice of treatment technique. Especially, patient with benign disease should be analysed very carefully according to this complexity of problems (for example dose distribution of patient 2 with a vestibularis schwannoma is illustrated in figure 5).

Higher cumulative dose in GTV as result of CAT can be subordinated in treatment for malignant diseases like metastases, but should be considered for irradiation of benign targets, too. Whenever OARs are involved in high dose areas risk of impairment with irreparable damage rises [17-20]. For example, treatment of vestibularis schwannoma involves the N. acousticus directly into the target volume. In this case higher cumulative doses using CAT should be considered. Similar results were achieved from Lagerwaard et al. 2009 [9]. In their work, RA irradiation for vestibular schwannomas was compared to conformal arc therapy. In conclusion, they found a better conformity and lower cumulative doses with equal dose exposure to the OAR and significant shorter treatment time for RA, too. These results had led to RA replacing CAT for vestibular schwannomas in their department.

In our study, treatment time was clearly shorter using RA for all patients. This fact results from fewer patient positioning procedures (1 time for RA; 1 time for every single arc using CAT) and single arc irradiation technique compared to several single arc using CAT. Whenever patient constitution allows no long recumbency, this parameter should be considered very carefully for choice of technique and could afford an important benefit for RA.

In this context, number of MU was clearly lower for RA in 8 of 10 patients. This item could be an advantage for patients because of less scattered radiation and for the daily routine of the department because of better time utilisation of the accelerators.

Interestingly, size of target GTV had no clearly impact on treatment choice in our patient population. For example, patients 1, 6 and 10 had a small GTV with a maximum of 0.3 cm^3 ^and were considered for RA treatment, whereas patient 3 with a GTV of 0.3 cm^3 ^was selected for CAT. In addition, in patients with larger GTVs (patients 4, 5, 8) no definitely benefit for one technique could be observed.

Doses at OAR were generally very low. Thus, this parameter should not be overestimated for technique selection but should be kept in mind especially for patients with exposure due to pre-irradiation of the brain or head. In these cases sparing of dose at OAR could be very important to avoid serious side effects during irradiation or in follow-up.

Comparing results of all parameters, choice of treatment techniques could be reclassified retrospectively in selected cases: patients 1, 5, 6, 7 and 9 achieved comparable dose exposure to the OAR for all measured parameters for both treatment techniques. Dose maximum, treatment time and number of MU showed clearly better results for RA. Solely, irradiated low dose volume was lower in patients 1, 5, 6 and 9 for CAT. Because of palliative indication, these five patients would have been treated with RA with much shorter treatment time and comparable OAR sparing, in future.

Patient 2 was irradiated because of a vestibularis schwannoma. In this case, dose to the brain stem and chiasm was higher using RA (figure 5). Furthermore, benign indication of irradiation increased the importance of the fact that involved low dose volume for RA was nearly twice as much as for CAT. An eventually higher risk of tumour induction by low dose irradiation areas [21-23] and higher OAR doses led to a final decision for CAT, although treatment time and number of MU were higher.

Analyzing patient 3 with two peripheral metastases led in a clear decision for CAT. The dose to all OAR, low dose volume and D_mean _of the healthy brain showed clearly better results for CAT. Merely, treatment time and number of MU would argue for RA but were reasonable for this patient using CAT.

For patient 8 CAT would be the treatment of choice, as well. Dose to the OAR and low dose volume were assessed as clear benefit for CAT, even though treatment time and number of MU were better using RA.

Results of patient 4 showed comparable results for both techniques. On the one hand, dose to OAR was slightly better using CAT for 4 of 6 items (table 2), but on the other hand, treatment time and dose maximum were better for RA. In the palliative situation of this patient, both choices of treatment technique should be arguable without any major disadvantages for this patient.

Analyses of patient 10 showed a retrospective decision in aid of RA. Shorter treatment time with less number of MU preponderated a slightly lower dose to the brain stem and chiasm as well as smaller low dose volume by use of CAT.

In summary, the choice of treatment technique should be done with respect to target entity, dimension and localisation as well as patient age and constitution. It is recommended to evaluate both techniques prior to treatment decision.

## Conclusion

We conclude that RA is a new approach for single fraction radiosurgery treatment. In selected cases, RA combines advantages of short treatment time with less number of MU and better conformity in addition to accuracy of stereotactic localisation, but with larger low dose areas in comparison to conventional cone based SRS. For this reason we successfully integrated this new approach into our treatment routine and started to irradiate patients with promising results.

Nevertheless, irradiation with CAT is not dispensable at the moment, but should rather kept in mind to be another feasible approach with different advantages for selected settings.

## Competing interests

The authors declare that they have no competing interests.

## Authors' contributions

All authors conceived of the study and participated in study design. HAW carried out the clinical evaluation and performed the statistical analysis. DMW and HV performed physical evaluation and technical implementing. HC and CFH worked in study coordination and helped to draft the manuscript. All authors read and approved the final manuscript.
